# Five Cases of Ovariotomy

**Published:** 1885-10

**Authors:** C. G. Davis

**Affiliations:** 240 Wabash Avenue


Five Cases of Ovariotomy. *By* C. G. Davis, m. d.

Few questions in surgery have so thoroughly occupied the attention of the profession, both at home and abroad., during the last twenty-five years, as those that arise in connection with the operation of ovariotomy. Some of these have been apparently decided, while many others still remain under discussion.

The rapid progress that has been made in the operation becomes marked when we note the successful results now constantly recorded, and then remember that no longer ago than 1861, the operation was spoken of as one "that would subject its performer to a criminal indictment for manslaughter." With our present successful methods, resulting in a constantly diminishing death-rate, we should not forget to give due praise and honor to those who have acted as pioneers in this work, and still to others who have led the advance in testing in the crucible of experience the various methods and plans of operating suggested. Looking back over their work we have been enabled to avoid their errors and to adopt that portion which seemed most successful.

The medical profession and the world in general will always owe a debt of gratitude to McDowell, Clay, Baker-Brown, Keith, Wells, Tait, Peaslee, Emmet and Atlee for the light they have shed on an operation which is without parallel in the advantages that have resulted to humanity.

In reciting the history of the following five cases, my entire experience in ovariotomy, it is not my object to boast of triumphs, but simply to perform what I consider a duty, in adding my experience to the great fund which has already been contributed to the subject.

Case I.—Mrs. T. W., Chicago, aged fifty years, consulted me at my office November 10, 1883. Her general health in previous years had been good, with the exception of headaches, from which she had only occasionally suffered. She began to menstruate in her sixteenth year, and this function had continued natural in every respect until she was forty-eight. At the time of the menopause she had noticed no unusual disturbance of her health. She was married at the age of twenty, and had given birth to eight children, the youngest being thirteen years old. About fifteen years ago she had twice suffered from miscarriages. Eighteen months previous to the time of her consulting me she began to notice a slight swelling, attended with some pain, in the region of the left ovary. She had been under the care of several physicians, and they had treated her for "dropsy."

On making a general examination I found her physical condition unusually good. Pulse, temperature, respiration and excretions were normal. Her face had the peculiar shrunken and discolored appearance indicative of ovarian disease. The abdomen was uniformly distended and appeared somewhat more enlarged than at the full time of pregnancy. Dullness and fluctuation over the entire surface. No bulging in the *cul-de-sac* of Douglas. Uterine sound showed the uterus to be normal as to size and position. Diagnosis, ovarian cyst.

Patient being averse to an operation for removal, I suggested tapping as a means of relief. I operated November 25th, on which occasion I was assisted by Dr. Plumb. I removed twenty pints of dark-colored fluid, resembling very much the appearance of strong coffee. The specific gravity was not taken. Chemical and microscopical examination revealed albumen, the ovarian cell, epithelium, etc. She was kept in bed a few days, was greatly relieved, and appeared to gain very much in flesh and strength. In a week the abdomen was noticed to be rapidly increasing in size. She called upon me to be relieved. I reluctantly consented, and on the 15th of January I again used the trocar and canula and withdrew about the same amount of fluid. The color was much brighter than before, being a light straw color. She did not derive as much benefit from this tapping as previously; in fact, her flesh and strength appeared to rapidly diminish.

In March she again called upon me at my office, seeking relief. I strongly urged the removal of the cyst. Requested her to call upon Dr. Dudley. He gave her a thorough examination, concurred in my diagnosis, and advised an immediate operation. She returned to me and consented. I selected her own home as the place for the operation. Full antiseptic measures were used in every respect. The room, furniture, clothes of the patient and bedding were all thoroughly carbolized with a three per cent. solution. She was sponged the night previous and the morning of the operation with a one per cent. solution. She was allowed a good, generous diet. The night before, a small dose of castor oil was administered, and a light breakfast of broth and tea was given at 8 A. M.

The operation was done on May 18th, with the assistance of Drs. Henry Plumb and M. S. Leech. Dr. Addison H. Foster administered the anæsthetic (ether).

An incision was made in the *linea alba*, beginning two inches below the umbilicus and extending downward three inches. The walls of the abdomen were thick and vascular, and considerable compression had to be used to stay the flow of blood. On entering the peritoneum about two ounces of ascitic fluid escaped. Extensive parietal adhesions were found which were broken down almost entirely with a No. 10 bougie. The cyst was punctured with a Spencer Wells trocar with rubber tube attached, and several pints of fluid drawn off. Several other smaller cysts appearing, into which it was difficult to introduce the trocar, the incision was enlarged, and with my hand I broke down the different septa and soon delivered the entire cyst through the opening. A number of omental adhesions were found which were easily separated; also about twelve inches of small intestines were found closely adherent to the under surface of the cyst, which were separated only by long and careful manipulation. The pedicle was about four inches broad, extending the whole length of the broad ligament and being attached to the left cornuel of the uterus. It was decided to tie it in two sections. Accordingly a needle, threaded with a double white silk cord, was passed through the center and each section tied, and the tumor excised. While holding the pedicle and applying the cautery, the ligature slipped from the larger section and immediately the abdomen began filling with blood. Grasping a pair of large forceps, I elevated the stump, procured another double thread and again tied both sections, the portion of the stump springing from the uterus being so short as to necessitate tying the ligature into the substance of the uterus itself. The cavity was thoroughly sponged, but fearing hæmorrhage from the stump, I placed a Thomas drainage tube in the lower angle of the wound extending to the *cul-de-sac* of Douglas. I closed the opening with six silk sutures. Carbolized oil, absorbent cotton and the flannel bandage completed the dressing. Duration of operation, one hour and a half. Five minims of Magendie's solution of morphine were administered hypodermically. I visited her at 6 P. M. and found good reaction, pulse 100, temperature normal.

She continued to progress favorably, with a temperature ranging from 100°F. to 100.5°F., and a pulse of 110, till the fifth day, when the temperature suddenly rose to 104°F. and the pulse to 140. She complained of severe pain in the region of the bladder, and a desire to pass her urine every half hour. I found her suffering from a severe cystitis, caused undoubtedly from too frequent introduction of the catheter. I washed out the bladder with a solution of boracic acid, ten grains to an ounce of lukewarm water, and ordered it repeated three times a day. In a few hours the temperature and pulse came to the usual average, and she progressed steadily to recovery. On the seventh day, there being no discharge through the drainage tube, I removed it. The stitches were removed gradually from the eighth to the tenth day. There was but slight suppuration at the stitch holes. She was up at the end of the third week, and is now in good health. Weight of cyst, together with contents, forty pounds.

CASE II.—Mrs. A. L. A., from Shelby, Neb., consulted me October 6th, 1884. She was twenty-eight years old and had a healthy family history. She began to menstruate at the age of fourteen, and had always been regular. She was married in her twentieth year and had given birth to two children, the youngest being five years old. Her previous general health had always been good till the last five years, during which period she had complained more or less of pain in the region of the right ovary, with occasional attacks of bloating in the abdomen. Her symptoms had never been severe, and she only consulted me at the earnest solicitation of friends. For two years there had been a gradual general enlargement of the abdomen, and at the time of the examination she presented appearance of being six months pregnant, The enlargement was symmetrical and fluctuation could be easily obtained. The uterus was normal as to size and position. No fluctuation could be found in the *cul-de-sac* of Douglas. No protrusion of the umbilicus. Diagnosis, ovarian cyst springing from the right ovary. In order to arrive at a more correct diagnosis I

introduced a large needle of a hypodermic syringe into the most prominent part of the abdomen, and withdrew about three drachms of fluid, which, on examination, presented the general appearance of the fluid of an ovarian cyst. I communicated the diagnosis to her sister who accompanied her. It was deemed best not to allow the patient to know of her condition then, as it was impossible for her to remain in the city and be operated on at that time. She returned home and continued to gradually enlarge. In November her physician wrote me that she was suffering from an attack of peritonitis. This continued for some three weeks, was very severe, and had it not been for the skill of her attending physician, Dr. J. A. Inks, she would undoubtedly have lost her life. I was earnestly solicited by her friends to come and perform the operation. I accordingly appointed December 19th. I found her still confined to her bed, not having entirely recovered her strength since the attack of peritonitis. She was much emaciated, the abdomen excessively distended, white and glistening; fluctuation could be obtained over the entire surface. Her home was situated in the suburbs of a prairie village—a veritable "cottage by the wayside," overlooking a stretch of prairie twenty miles in extent. All of the hygienic surroundings were of the most promising nature, but notwithstanding this I adhered strictly to all of the antiseptic measures, using the carbolic spray and seeing that all the attendants, instruments, etc., were thoroughly carbolized.

The patient was allowed her usual diet up to the day of the operation, when she breakfasted on milk porridge and a cup of tea at 7 A. M. Bowels had been moved the previous day with castor oil. Operation was begun at 10 A. M. Patient was etherized by Dr. F. Englehard. I was assisted by Drs. G. H. Peebles, T. W. Hayden, East, and J. A. Inks.

An incision was made in the *linea alba* three inches in length. There were no parietal adhesions. The cyst was tapped and the fluid evacuated without a drop escaping into the abdominal cavity. On withdrawing the cyst a slight omental adhesion was discovered. The pedicle was long and about an inch and a half broad. It was transfixed and tied with a No. 17 iron-dyed Chinese silk cord, the tumor excised and the pedicle seared with a common cautery iron heated in the fire. The abdomen was thoroughly sponged and the opening closed with four silk sutures. Carbolized dressing was applied, the patient put to bed, and five minims of Magendie's solution of morphine given hypodermically.

In thirty minutes I was compelled to leave her and take my departure for home. Before I left she had reacted and expressed herself as feeling quite well. Dr. Inks wrote me subsequently and sent me the following record of her pulse and temperature :

| | | | | | |
|---|---|---|---|---|---|
| Dec. 20th, 1 P. M.—Temp., | | 97.5°F. | Pulse, | 120. |
| " " 7 " | " | 99. | " | 112. |
| " 21st, 7 A. M. | " | 98.5. | " | 130. |

After this date the temperature was normal and pulse less rapid. The wound healed without suppuration and the sutures were removed on the eighth day. Her recovery was rapid and her health since the operation is reported much better than for many years. Weight of tumor estimated at twenty-five pounds.

CASE III.—Mrs. C. S., Chicago, aged thirty-seven, consulted me April 10th, 1885. Her general health had not been good since the birth of her only child, thirteen years ago. Had suffered much from headache, constipation and general debility. Menstruation began at the age of sixteen years, was quite regular, but always more or less painful. She was married in her twenty-second year. Three years ago she was troubled with difficult micturition, and the obstruction became so great as to necessitate having the urine drawn with a catheter. At that time she consulted Dr. Byford, who told her she had a tumor, gave her medical treatment, but advised against an operation. At the time of her consulting me I found her much emaciated, countenance discolored and her strength very feeble. Abdomen was large, evenly distended, very tense, elastic and showed dullness and fluctuation over the entire surface. The growth had begun three and a half years ago, when she noticed an enlargement in the region of the left ovary. Recently she had increased in size very rapidly, and earnestly requested that I operate and relieve her. The uterus seemed to be slightly enlarged and anteverted, but the sound failed to enter more than two inches. From the history of the case and the present symptoms, I had no hesitation in diagnosticating ovarian cyst.

Selecting an upper room in her own home, I advised her to have the operation there, thinking it would be safer, from a hygienic point of view, than the wards of a public hospital. The operation was done April 7th, 1885. I was ably assisted by Dr. Henry Plumb; present, Mr. C. B. Reed, medical student. Dr. King administered the ether. Antiseptic measures were used throughout the operation.

An incision was made in the *linea alba*, beginning two inches below the umbilicus and extending downward three inches. A few slight adhesions were encountered, which were readily overcome, the cyst punctured, and several pints of straw-colored fluid withdrawn. The uterus was discovered to be much hypertrophied, extending upward into the abdominal cavity some four inches and a half, and being over three inches in breadth. Being pressed between the abdominal wall and the cyst had caused it to become excessively flattened. Had I succeeded in introducing the sound to the full extent of the uterus, I should certainly have hesitated to operate, The narrowness of the cervix, together with the extensive anteversion, had prevented this. It was this pressure on the bladder, undoubtedly, that had so often caused retention of the urine. The pedicle was two inches broad. I secured it with Chinese silk cord No. 17, using the "Staffordshire" knot of Lawson Tait. The cyst was excised and the stump seared thoroughly with Paquelin's thermo-cautery.

The loss of blood being slight, but little difficulty was experienced in "making the toilet of the peritoneum." The incision was closed by four silk sutures, and the wound dressed with carbolized oil, cotton batting, and the flannel bandage applied. Reaction came on well in an hour and a half. Five minims of Magendie's solution of morphine were given hypodermically. Patient continued to do well; temperature not running over 101°F. and pulse ranging from 100 to 110 till the fourth day, when dysenteric discharges began, accompanied with rapid distension of the abdomen with gas. The distension was noticed in the morning, and by evening it had become so great as to open slightly the lower angle of the wound, and threatened to re-open the entire wound. The temperature ran to 102.5°F, and the pulse to 130, yet there were no symptoms of peritonitis. Remembering that this sudden accumulation of gas in the intestine was considered a dangerous symptom by Lawson Tait, I of course regarded it seriously. Recognizing that it must come from decomposition going on in the intestinal canal, I gave:

℞. Sodii Sulphitis .................... ʒ i.
Tinct. Card. Comp...............q. s. ad. f. ℥ iij.
M. S. Teaspoonful every three hours in water.

On the fifth day the symptoms had almost entirely subsided, and pulse and temperature were again reduced. She improved gradually but slowly, the temperature vibrating from 100° to 101.5°F. and the pulse from 90 to 105 till the end of the fourth week, when both became normal. The cyst was multilocular, and with the contents weighed twenty-three pounds.

She has recently called upon me and expressed herself as enjoying better health than ever.

CASE IV.—Mrs. R. R. B., of Waterman, Ill., aged twenty-six, first became my patient September 15th, 1882. Menstruation had occurred at the age of fourteen, had always been painful, and it was for this ailment that she consulted me. She had been married three years and had never been pregnant. She was extremely nervous, and this symptom was constantly exaggerated from the shock she received from pain experienced at each menstrual period. On making an examination I found tenderness of the right ovary and slight symptoms of enlargement. I gave her a variety of treatment, which seemed for the time being only to be palliative. The dysmenorrhœa would apparently subside for a month or so and then return in its severity. She continued in this variable condition for a year or so. In November, 1884, she returned to me. I found the abdomen enlarged to the size of the third month of pregnancy. Examination showed the uterus to be normal. Abdomen much enlarged on right side. Deep pressure could detect an enlargement the size of a large goose egg in the region of the right ovary, in which I could readily distinguish fluctuation by pressing simultaneously in the vagina and on the outer surface of the abdomen. My diagnosis was an ovarian cyst, with the advice that she have an immediate operation. She and her friends shrank from this ordeal. She consulted a "Magnetic Physician," who said "he could cure her, having rubbed away several of the same kind." In the latter part of May, of the present year, she again called upon me. I found the abdomen much enlarged and general fluctuation. She now consented to the operation, and I appointed June 7th, at 12 A. M., as the time, and selected for her large, well-ventilated rooms in a private residence in the city. I was ably assisted by Dr. Plumb—present, also, Dr. Annette S. Dobbin and Mr. Reed, medical student. Dr. King administered the ether.

The incision was made in the usual region and three inches in length. There were no adhesions. The walls of the abdomen were very thick and vascular, but the hæmorrhage was easily controlled with pressure forceps. On tapping the cyst I withdrew several pints of liquid, and on removing the trocar a quantity of dermoid material made its appearance. It was evidently a dermoid cyst, containing quite a quantity of fatty material and hair. The pedicle was an inch and a half in breadth. I used the "Staffordshire" knot, with Chinese silk No. 17, and excised the cyst, searing the stump with Paquelin's thermo-cautery. I do not think that more than a tablespoonful of blood was lost in the entire operation. The abdomen was closed with four silk sutures, and the usual dressing applied. Pulse 120, and temperature 99°F.

6 P. M.—Pulse 140°F., temperature 100°F., weight of cyst 18 pounds. For the first week temperature ranged from 99°F. to 101°F., and pulse continued from 115 to 130. She was very nervous, restless, and obtained but little sleep. Sedatives and hypnotics seemed to have but little effect. She had never been able to take opiates of any kind. I gave morphine hypodermically, which only aggravated her nervousness. Her restlessness was best quieted by sponging the temples with warm water, which was done every hour or so. Pulse and temperature became normal in the third week, after which she rapidly gained strength and is now quite well, the dysmenorrhœa being entirely relieved.

Case V.—Mrs. S. C. T., Chicago, consulted me June 22, 1885. She was thirty-six years of age. Menstruation began at the age of fourteen, and had always been regular and free from pain till the beginning of her present trouble. When twenty-three years old she was married, had given birth to two children, the youngest being ten years of age. Previous to the present disease her health had always been good. She dated the beginning of her illness from January, 1884. She complained of slight pain, pressure or heaviness in the region of the right ovary, and in about two months after that the abdomen was observed to be growing larger. For some time she was treated by her family physician, who considered her to be pregnant till the lapse of several months, when he told her she was suffering from a morbid growth and counseled her to seek surgical advice. For several months previous to her calling upon me she had been treated by manipulation from a "magnetic physician." The last three menstrual periods had been irregular and the flow much diminished. There was no œdema of the lower extremities. Recently the distention had seemed to increase much more rapidly than before. The abdomen appeared to be uniform in shape, soft, elastic, and over the entire surface fluctuation could be obtained. On making a deep and sudden pressure with the hand below the umbilicus a tumor could be easily detected, which floated in the surrounding fluid and could be easily displaced from one side of the abdomen to the other. The sound, when introduced into the uterus, entered to the extent of two and a half inches. Movement of the tumor did not seem to materially affect the uterus.

Percussion gave resonant sounds in the umbilical region, while the surrounding surface was dull.

My diagnosis was ovarian tumor, probably complicated with ascites. I advised an immediate operation. Patient shrank from the ordeal and decided to defer, at least for the present, any surgical treatment. On the 30th of July she again con-consulted me. She had increased in size several inches and was much emaciated. Using the same antiseptic precautions as in the previous cases, the operation was done at her residence, August 2, 1885. I was assisted by Drs. Anna Thomas and Henry Plumb. The anæsthetic was administered by Dr. King, Present, Mr. Reed, medical student.

An incision three inches long was made in the usual region, below the umbilicus, and the various tissues divided down to the peritoneum. The walls of the abdomen were very thin and but little hæmorrhage occurred. Immediately upon making a slight opening into the peritoneum, a thick, dark-colored, colloid-appearing fluid gushed out, showing the great tension of the abdominal walls. This fluid resembled so closely the contents of other ovarian cysts I had seen, that I was for the moment fearful that there might be some error of diagnosis and that I had entered the cyst itself. But on making further examination, by introducing the finger deeply through the opening, the tumor could be easily felt floating in the abdominal cavity. The incision through the peritoneum was now enlarged to an extent equal to that of the other structures, and the abdominal cavity emptied of the fluid. The trocar was then introduced into the tumor, but no fluid was obtained save a few drops of thick gelatinous material, which was evidently too viscid to flow. The trocar was then withdrawn, the incision extended to the umbilicus, and I introduced my hand, breaking down the central structure of the mass. I finally drew it through the opening. There were no adhesions. The pedicle was about two inches broad, springing from the right ovary and broad ligament. It was secured by the "Staffordshire knot" of Chinese silk, No. 17, the tumor excised and the stump well seared with Paquelin's cautery.

The "toilet of the peritoneum" was thoroughly done with water, at 98°F., till the current returned nearly or quite colorless. The abdomen was closed with six interrupted silk sutures, and remembering the large quantity of peritoneal fluid, and thinking that more might be secreted, a Thomas drainage tube was introduced into the lower angle of the wound, extending to the *cul-de-sac* of Douglas. The usual carbolized dressing was applied. Symptoms of collapse being manifest, brandy was given hypodermically, and the patient placed in bed surrounded with bottles of hot water. In about half an hour reaction took place. As the patient was restless and seemed inclined to vomit, a hypodermic injection of morphine was administered.

The operation was completed at 11 A. M., just one hour having elapsed since the first incision was made. On visiting the patient again at 6 P. M., I found the pulse 108°F. and temperature 99°F. She had vomited once at 2 P. M., but otherwise had rested quietly. One tablespoonful of milk was given every two hours, which seemed to agree well with the stomach. On the next day, 8 A. M., pulse was 105 and temperature 101°F. She complained much of pressure or pain in the region where the drainage tube entered the abdomen I removed the bandage, found the wound in an apparently good condition, and, as there was no appearance of drainage through the tube, I removed it.

August 4th, 7 A. M.—Pulse 117 and temperature 108.8°F. Stomach in good condition and patient craving food.

August 5th.—Pulse 105 and temperature 100.8°F. Nurse stated that the dressing appeared saturated with discharge from the wound. On removing the bandage I found the abdomen slightly distended and a bloody serum oozing through the lower angle of the incision.

On making a slight pressure I caused the discharge of something like an ounce of fluid, and then again applied the dressing.

August 6th, 8 A. M.—Pulse 100 and temperature 99°F. Serum still discharging. Applied fresh carbolized dressings.

August 7th, 8 A. M.—Pulse 105 and temperature 100.8. Patient very restless. Bandage was saturated with the discharge, which gave a strong odor. I removed the dressing, and taking a No. 10 Nelaton,s catheter I introduced it through the lower angle of the wound several inches into the abdominal cavity, and using a common hand syringe I pumped out two ounces of very offensive pus. Using a solution of five grains of carbolic acid and ten grains of chloride of sodium to a pint ot water, at a temperature of 98°F., I washed out the abdominal cavity till the water returned clear and odorless. Left the catheter in position.

Aug. 8th, 8 A. M.— Pulse 100 and temperature 100.4°F. Patient had slept well and expressed herself as feeling much better since the operation of washing out the abdomen the evening before. Found pus oozing from the catheter and the wound. Applied the syringe and withdrew an ounce of pus and washed out the cavity. At 8 P. M., pulse 95 and temperature 100°F. Again withdrew an ounce of pus and repeated the cleansing process. Removed the catheter and used instead a glass drainage tube, which I had myself made from a common glass tube, bending it in a spirit flame to the shape required. In a few days adhesions formed and an artificial wall seemed to be growing, so that the injection did not pass over the entire cavity of the abdomen, and each day it seemed to contain less fluid. This process of cleansing the abdominal cavity was repeated twice a day for two weeks, till August 27th, when the cavity was so far healed as to admit the drainage tube only an inch, and it was removed.

At the time of making this report all discharge has ceased, and but a slight abrasion remains in the location of the wound. Weight of cyst and fluid estimated at thirty pounds.

A subsequent examination of the cyst showed several small openings. Through these, undoubtedly, the fluid had escaped, and so caused the accumulation in the abdominal cavity.

From a careful review of the above five cases of ovariotomy, I am led to the following conclusions:

1st. That success, however much it may depend upon any mode or plan of operating, is also largely controlled by strict attention to the minutiæ which relates to the patient and the surroundings.

2nd. That thorough cleanliness is a great desideratum, and is best insured by a close adherence to what we call "antiseptic precautions."

3rd. That while recognizing the wonderful absorbing power of the peritoneum, we should be encouraged, in the intra-peritoneal method, to entirely close the abdominal incision. Still, this absorbing tendency may be impaired or destroyed by the long continuance of fluid in the abdominal cavity, and in these cases drainage is indispensable.

4th. That an opiate of some kind, possibly morphine hypodermically administered shortly after the operation, fortifies the nerve centers, brings on better reaction, and by quieting peristaltic action of the intestines, gives rest to the wounded parts, and in this manner hastens and assists to a more rapid start towards recovery.

240 Wabash Avenue.

